# On noise-resolution uncertainty in quantum field theory

**DOI:** 10.1038/s41598-017-04834-y

**Published:** 2017-07-03

**Authors:** Timur E. Gureyev, Alexander Kozlov, Yakov I. Nesterets, David M. Paganin, Harry M. Quiney

**Affiliations:** 1 ARC Centre in Advanced Molecular Imaging, School of Physics, The University of Melbourne, Parkville, 3010 Australia; 2 School of Physics and Astronomy, Monash University, Clayton, 3800 Australia; 3 School of Science and Technology, University of New England, Armidale, 2351 Australia; 4 CSIRO Manufacturing, Clayton, 3168 Australia

## Abstract

An uncertainty inequality is presented that establishes a lower limit for the product of the variance of the time-averaged intensity of a mode of a quantized electromagnetic field and the degree of its spatial localization. The lower limit is determined by the vacuum fluctuations within the volume corresponding to the width of the mode. This result also leads to a generalized form of the Heisenberg uncertainty principle for boson fields in which the lower limit for the product of uncertainties in the spatial and momentum localization of a mode is equal to the product of Planck’s constant and a dimensionless functional which reflects the joint signal-to-noise ratio of the position and momentum of vacuum fluctuations in the region of the phase space occupied by the mode. Experimental X-ray synchrotron measurements provide an initial verification of the proposed theory in the case of Poisson statistics.

## Introduction

A cornerstone of quantum mechanics is the fact that certain quantities, like position and momentum of a particle, cannot be measured simultaneously with arbitrary precision. Similarly, quantum field theory sets a limit for the precision of certain measurements due to the presence of vacuum fluctuations. The two phenomena are not equivalent, but they are related. In the present work we establish a quantitative relationship between these two aspects of quantum measurement, which has the form of a noise-resolution uncertainty inequality involving the variance of the position or momentum of the field’s mode on one hand and the variance of the energy of the mode on the other. We show that the product of the two variances can never be smaller than a certain absolute positive constant. Importantly, the uncertainty relationship derived here is qualitatively different from the classical Heisenberg uncertainty^1–3^, as it reaches its minimum not for Gaussian distributions or coherent states, but for the so-called Epanechnikov distributions (which have the form of a truncated inverted parabola) that are well known in mathematical statistics^4–6^. This particular fact distinguishes our uncertainty relationship from the number-phase uncertainty^7^. There are several other known uncertainty relationships related to the Heisenberg uncertainty^8–15^. In particular, the Robertson uncertainty principle^8^ represents a generalized form of the Heisenberg uncertainty. In turn, the Robertson uncertainty follows directly from the Schrödinger uncertainty inequality^9^. As far as the physical meaning of the Heisenberg uncertainty is concerned, different interpretations have been discussed, starting from Heisenberg’s own interpretation^1^ and the similar interpretation by Feynman^2^. In particular, the difference between the disturbance to the state of the system produced by the measurement (“systematic errors”) and the variance in the measured quantities as a result of “statistical imprecision” of the measurements have been analysed as different sources contributing to the overall uncertainty in the measurements^10, 12^. Reviews of the history of the subject and related concepts can be found in^3, 10, 14^.

As a motivating example for this Report, consider a three-dimensional volume of space which is permeated by a quantised electromagnetic field. Suppose that the number of photons which will be extracted from this field is a specified integer, and that the volume over which photon detection is to take place is also fixed. In building up an intensity map of localisable features in the photon field, there is an evident practical trade-off between noise and resolution; increasing the number of spatial resolution elements (“voxels”) in the fixed specified volume will increase resolution (i.e. decrease voxel size) of the detected intensity signal, at the expense of increased relative noise in the intensity signal. The first key result of this Report is a noise-resolution uncertainty principle which quantifies this trade-off by establishing a lower bound on the product of the spatial resolution volume and the variance of the intensity signal. Note that localization of a field can be achieved either by the use of a detector with sufficient spatial resolution or by considering fields which contain a single spatially-localized feature. For the purposes of our study, the two approaches are essentially equivalent and in the present Report we adopt the latter.

The noise-resolution uncertainty principle reported here is neither reducible to, nor derivable from Heisenberg’s uncertainty principle. Moreover, when applied to both the uncertainty of the momentum and position of a boson field, it leads to a modified form of the Heisenberg uncertainty inequality in which the reduced Planck constant on the right-hand side is multiplied by a dimensionless functional reflecting the signal-to-noise ratio (SNR) of the momentum and position of vacuum fluctuations in the region of the phase space occupied by the mode. When the value of this functional is equal to unity, the modified inequality reduces to the conventional Heisenberg uncertainty inequality. However, when the value of the functional is large, our modified inequality predicts that the minimum of the product of uncertainties of the momentum and position of the field can actually be larger than the one predicted by the Heisenberg uncertainty. The additional uncertainty is associated with the trade-off between the precision of a simultaneous measurement of position and momentum, and the SNR of these measurements. We emphasize that (a) neither of the above two cases violate the Heisenberg uncertainty principle, (b) more interestingly, the latter case gives a circumstance in which the Heisenberg uncertainty is not sufficiently strong, i.e. circumstances in which the right hand side of the Heisenberg principle can be multiplied by a number larger than unity and still remain true.

The trade-off between spatial resolution and SNR is important for many imaging and communication problems, where simultaneous optimization of both spatial resolution and SNR is usually desired^16, 17^. A similar trade-off between noise and spatial resolution can in fact be demonstrated in the case of some fundamental experiments involving quantum measurements, such as Young’s double-slit experiment with electrons^2, 18, 19^. The key results of the present paper all amount to a quantitative study of the form of this well-known trade-off, in the context of a quantised electromagnetic field theory.

## Results

### Noise-resolution uncertainty inequality for boson fields

Consider a linearly-polarized quantised electromagnetic field defined by an operator-valued distribution $E({\bf{x}})=\sum _{k}{E}_{k}({\bf{x}})$, where ${E}_{k}({\bf{x}})$ = $i{(\hslash {\omega }_{k}/2)}^{1/2}[{u}_{k}({\bf{r}})\exp (-i{\omega }_{k}t){a}_{k}$ − ${u}_{k}^{\ast }({\bf{r}})\,\exp (i{\omega }_{k}t){a}_{k}^{\dagger }]$, **x** = (**r**, *t*) is a four-dimensional space-time point, *ħ* is the reduced Planck constant, *ω*_*k*_ are the angular frequencies of the modes, *u*_*k*_(**r**) are the mode functions, *a*_*k*_ and ${a}_{k}^{\dagger }$ are the photon annihilation and creation operators, respectively^7, 20^. We assume the mode functions *u*_*k*_(**r**) to be orthonormal, in the usual sense that $\int d{\bf{r}}\,{u}_{{k}_{1}}^{\ast }({\bf{r}}){u}_{{k}_{2}}({\bf{r}})={\delta }_{{k}_{1}{k}_{2}}$, where ${\delta }_{{k}_{1}{k}_{2}}$ is the Kronecker symbol, but we do not limit the choice of these functions only to (truncated) plane waves, so the index *k* here is just a label indexing a complete set of modes^20^. We study the behaviour of the time-averaged space-integrated variance of the field’s intensity, (1/2)*E* ^2^(**r**, *t*), for a state |*ψ*〉 of the field:

1 $${({\rm{\Delta }}{I}_{\psi })}^{2}\equiv \mathop{\mathrm{lim}}\limits_{T\to \infty }\frac{1}{T}{\int }_{-T/2}^{T/2}dt\int d{\bf{r}}(1/4)[\langle \psi |{E}^{4}({\bf{r}},t)|\psi \rangle -{(\langle \psi |{E}^{2}({\bf{r}},t)|\psi \rangle )}^{2}].$$

We are primarily interested in the states of the field with small numbers of photons, where the effect of vacuum fluctuations can be prominent. In such cases it is often convenient to work with the Fock space representation. Using the standard properties of the Fock states, |*n*〉, and the field creation and annihilation operators^7^, it can be shown by direct calculation that, for a single-mode case, one has:

$$\begin{array}{c}\langle n|{E}_{k}^{2}({\bf{x}})|n\rangle =\hslash {\omega }_{k}(n+1/2){|{u}_{k}({\bf{r}})|}^{2},\\ \langle n|{E}_{k}^{4}({\bf{x}})|n\rangle =(3/2){(\hslash {\omega }_{k})}^{2}[{(n+1/2)}^{2}+1/4]{|{u}_{k}({\bf{r}})|}^{4},\end{array}$$

and hence,

2 $$\begin{array}{rcl}{({\rm{\Delta }}{I}_{n})}^{2} & = & (1/4)\int d{\bf{r}}[\langle n|{E}_{k}^{4}({\bf{x}})|n\rangle -{(\langle n|{E}_{k}^{2}({\bf{x}})|n\rangle )}^{2}]\\  & = & [{(n+1/2)}^{2}+3/4]\,[{(\hslash {\omega }_{k})}^{2}/8]\int d{\bf{r}}{|{u}_{k}({\bf{r}})|}^{4}\ge {({\rm{\Delta }}{I}_{0})}^{2},\end{array}$$

where ${({\rm{\Delta }}{I}_{0})}^{2}=[{(\hslash {\omega }_{k})}^{2}/8]\int d{\bf{r}}|{u}_{k}({\bf{r}}){|}^{4}$ is the intensity variance of the vacuum state |0〉. Note that it is essential that we are considering the variance of the field’s intensity (energy) here, rather than the variance of the photon number, as the latter quantity would be zero for Fock states. Using a conventional variational approach, it is also possible to show that the variance of field intensity in any state cannot be smaller than that of a vacuum state. As an example, explicit calculations for the intensity variance in a (Glauber) coherent state |*α*〉^7, 20^, labelled by a complex number *α*, produce:

3 $$\begin{array}{rcl}{({\rm{\Delta }}{I}_{\alpha })}^{2} & = & \mathop{\mathrm{lim}}\limits_{T\to \infty }\frac{1}{T}{\int }_{-T/2}^{T/2}dt\int d{\bf{r}}(1/4)\,[\langle \alpha |{E}_{k}^{4}({\bf{x}})|\alpha \rangle -{(\langle \alpha |{E}_{k}^{2}({\bf{x}})|\alpha \rangle )}^{2}]\\  & = & ({|\alpha |}^{2}+1/2)[{(\hslash {\omega }_{k})}^{2}/4]\int d{\bf{r}}{|{u}_{k}({\bf{r}})|}^{4}\ge {({\rm{\Delta }}{I}_{0})}^{2}.\end{array}$$

Notice that, because the mode functions are normalized in the usual sense that $\int d{\bf{r}}{|{u}_{k}({\bf{r}})|}^{2}=1$, a narrow (tightly localized) mode function will correspond to large intensity variance in Equations (2) and (3). This behaviour can be easily seen in the example of a truncated plane wave, ${u}_{k}({\bf{r}})={L}^{-3/2}\exp (i{\bf{k}}\cdot {\bf{r}}){\chi }_{L}({\bf{r}})$, where *χ*_*L*_(**r**) is equal to one inside a cube *V*_*L*_ centred at **r** = 0, with sides of length *L* parallel to the coordinate axes, and *χ*_*L*_(**r**) is equal to zero outside the cube. Here $\int d{\bf{r}}|{u}_{k}({\bf{r}}){|}^{2}={L}^{-3}\int d{\bf{r}}{\chi }_{L}({\bf{r}})=1$ and ${({\rm{\Delta }}I)}^{2}\propto \int d{\bf{r}}{|{u}_{k}({\bf{r}})|}^{4}={L}^{-3}\mathop{\longrightarrow }\limits_{L\to 0}\infty$. The well-known observation that the variance of the field intensity increases with increased spatial localization is made quantitative in a general context by the “noise-resolution uncertainty principle” described below.

We define the spatial width of a mode as

4 $${({\rm{\Delta }}r)}^{2}=\int d{\bf{r}}|{\bf{r}}-\bar{{\bf{r}}}{|}^{2}|{u}_{k}({\bf{r}}){|}^{2},$$

where $\bar{{\bf{r}}}$ is the mean value of variable **r** with probability density function |*u*_*k*_(**r**)|^2^. The following mathematical inequality is a special case of the main result from^6^:

5 $$\int d{\bf{y}}|f({\bf{y}}){|}^{4}[(4\pi /d)\int d{\bf{y}}|{\bf{y}}-{{\bf{y}}}_{0}{|}^{2}|f({\bf{y}}){|}^{2}]{}^{d/2}\,\ge \,{C}_{d},$$

where *d* is any positive integer, *f*(**y**) is a function defined on a *d-*dimensional vector space, normalised such that $\int {\bf{dy}}{|f({\bf{y}})|}^{2}=1$, **y**_0_ is an arbitrary constant vector, *C*_*d*_ = 2^*d*^Γ(d/2)*d*(*d* + 2)/(*d* + 4)^d/2+1^ is a positive constant which depends only on *d*, and Γ(·) is the Gamma function. The equality in Equation (5) is achieved for truncated inverted parabola (Epanechnikov) distributions |*f*_*E*_(**y**)|^2^ = *c*_1_(1 − |*c*_2_ **y** − **y**_0_|^2^)_+_, where *c*_1_ and *c*_2_ are arbitrary positive constants, and the subscript “+” denotes that the function is equal to zero for those values of its argument, where the expression inside the brackets is negative^6^. Taking *d* = 3 and *f*(**r**) = *u*_*k*_(**r**), we obtain from Equations (2–5) the first key result of this Report:

6 $${({\rm{\Delta }}r)}^{3}{({\rm{\Delta }}{I}_{\psi })}^{2}/{W}_{0}^{2}\ge 2(\int d{\bf{r}}{|{u}_{k}({\bf{r}})|}^{4}){(\int d{\bf{r}}{|{\bf{r}}-\bar{{\bf{r}}}|}^{2}|{u}_{k}({\bf{r}}){|}^{2})}^{3/2}\ge {C}_{3}^{^{\prime} }$$

where *W*_0_ = *ħω*_*k*_/4 is the energy of the vacuum state of the electric field and ${C}_{3}^{^{\prime} }={(3/\pi )}^{3/2}{C}_{3}/4$ = $15\sqrt{27}/({7}^{5/2}\pi )\cong 0.19$ is a dimensionless constant. This inequality expresses an uncertainty relationship that exists between the noise in the measured intensity of a field mode and the variance of its spatial distribution which quantifies the degree of its spatial localization. Using Equation (5), the noise-resolution uncertainty relationship can be easily generalized to arbitrary (integer) dimension *d* = 1, 2, 3, …. The case *d* = 2, for example, can be applied to cross-sections of beams in planes orthogonal to the optical axis.

It is possible to re-formulate Equation (6) in terms of the spatial averages of the intensity, ${I}_{L,\psi }$ = $\mathop{\mathrm{lim}}\limits_{T\to \infty }\frac{1}{T}{\int }_{-T/2}^{T/2}dt\frac{1}{{L}^{3}}{\int }_{{V}_{L}}d{\bf{r}}(1/2)\langle \psi |{E}_{k}^{2}({\bf{x}})|\psi \rangle$, and the intensity variance, ${({\rm{\Delta }}{I}_{L,\psi })}^{2}$ = $\mathop{\mathrm{lim}}\limits_{T\to \infty }\frac{1}{T}{\int }_{-T/2}^{T/2}dt\frac{1}{{L}^{3}}{\int }_{{V}_{L}}d{\bf{r}}(1/4)$ $[\langle \psi |{E}_{k}^{4}({\bf{x}})|\psi \rangle$ − ${(\langle \psi |{E}_{k}^{2}({\bf{x}})|\psi \rangle )}^{2}]$, over a cube *V*_*L*_. In this case the noise relative to vacuum, (Δ*I*_*L*,*ψ*_)^2^/*I* ^2^_*L*,0_, is dimensionless, while the spatial resolution volume (Δ*r*)^3^ is replaced by its relative value, (Δ*r*)^3^/*L* ^3^, which can be interpreted as the fraction of the cube’s volume occupied by the mode. As a result, an analogue of Equation (6) can be written as:

7 $$\frac{{({\rm{\Delta }}r)}^{3}}{{L}^{3}}\frac{{({\rm{\Delta }}{I}_{L,\psi })}^{2}}{{I}_{L,0}^{2}}\ge {C}_{3}^{{\rm{^{\prime} }}}.$$

The limit of this inequality at *L* → ∞ coincides with Equation (6).

To appreciate the behaviour of Equation (6), again consider the case of a truncated plane wave, ${u}_{k}({\bf{r}})={L}^{-3/2}\exp (i{\bf{k}}\cdot {\bf{r}}){\chi }_{L}({\bf{r}})$, in a Fock state |*n*〉 or in a coherent state |*α*〉. From Equations (2–4) we obtain: (Δ*r*)^3^ = (*L*/2)^3^, ${({\rm{\Delta }}{I}_{n})}^{2}/{W}_{0}^{2}=2[{(n+1/2)}^{2}+3/4]{L}^{-3}$ and ${({\rm{\Delta }}{I}_{\alpha })}^{2}/{W}_{0}^{2}=4({|\alpha |}^{2}+1/2){L}^{-3}$. Clearly, here the values of the spatial resolution and the relative noise are counter-balanced with respect to the parameter *L*, with the expression in Equation (6) being equal to ${({\rm{\Delta }}r)}^{3}{({\rm{\Delta }}{I}_{n})}^{2}/{W}_{0}^{2}=[{(n+1/2)}^{2}+3/4]/4\ge 1/4 > {C}_{3}^{{\rm{^{\prime} }}}$ and ${({\rm{\Delta }}r)}^{3}{({\rm{\Delta }}{I}_{\alpha })}^{2}/{W}_{0}^{2}=({|\alpha |}^{2}+1/2)/2\ge 1/4 > {C}_{3}^{{\rm{^{\prime} }}}$, respectively. One can also see that when the photon energy statistics are known, inequalities (6) and (7) can sometimes be considerably strengthened. For example, if the photon energy statistics are Gaussian, as in the case of Fock states, then it can be seen that the ratio ${({\rm{\Delta }}{I}_{n})}^{2}/{W}_{n}^{2}=(1/2)[1+3{(2n+1)}^{-2}]\int d{\bf{r}}{|{u}_{k}({\bf{r}})|}^{4}$, where *W*_*n*_ = *W*_0_(2*n* + 1) is the energy of the mode in state |*n*〉, quickly approaches a constant asymptote when *n* increases. As a result, Equation (6) can be replaced by a stronger inequality: ${({\rm{\Delta }}r)}^{3}{({\rm{\Delta }}{I}_{n})}^{2}/{W}_{n}^{2}\ge {C}_{3}^{{\rm{^{\prime} }}}/4$. If the photon statistics are Poissonian, as in the case of coherent states, then ${({\rm{\Delta }}r)}^{3}{({\rm{\Delta }}{I}_{\alpha })}^{2}/{W}_{\alpha }^{2}\ge {C}_{3}^{{\rm{^{\prime} }}}/(2\bar{n}+1)$, where *W*_*α*_ = *W*_0_(2|*α*|^2^ + 1) and $\bar{n}={|\alpha |}^{2}$. Consequently, Equations (6) and (7) can be modified in the general *d-*dimensional case to give the following noise-resolution uncertainty principle:

8 $$\mathop{\mathrm{lim}}\limits_{L\to \infty }\frac{1}{{M}_{L}SN{R}_{L,\psi }^{2}}\equiv \mathop{\mathrm{lim}}\limits_{L\to \infty }\frac{{({\rm{\Delta }}r)}^{d}}{{L}^{d}}\frac{{({\rm{\Delta }}{I}_{L,\psi })}^{2}}{{I}_{L,\psi }^{2}}={({\rm{\Delta }}r)}^{d}\frac{{({\rm{\Delta }}{I}_{\psi })}^{2}}{{W}_{\psi }^{2}}\ge \frac{{C}_{d,\gamma }}{{(\bar{n}+1/2)}^{\gamma }},$$

where *M*_*L*_ = *L* ^*d*^/(Δ*r*)^*d*^ is the number of spatial resolution units in the measuring system, *SNR*_*L,ψ*_ =* I*_*L*,*ψ*_/Δ*I*_*L*,*ψ*_ is the average SNR, ${W}_{\psi }={W}_{0}(2\bar{n}+1)$, *C*_*d*,*γ*_ is a positive constant which depends only on the dimensionality of the space and the photon energy statistics, and *γ* = 0,1 or 2, respectively, in the case of Gaussian statistics, Poissonian statistics and generic statistics (which corresponds to Equation (6) and is valid in all cases, including sub-Poissonian statistics, in particular). In words, Equation (8) makes precise the statement that forms a core theme of the present paper, namely the fact that increasing the number of spatial resolution elements must be traded off against a corresponding reduction of SNR in each of these elements, when the mean number of detected photons is fixed. Compared to the more general, but more abstract, Equation (6), the last result has two potential advantages in that (a) it is formulated in terms of dimensionless and intuitively obvious quantities, and (b) it defines different lower limits for the noise-resolution uncertainty, depending on the energy statistics of the multi-photon states.

In the case of Poissonian statistics (*γ* = 1), Equation (8) is close in form to the noise-resolution uncertainty previously demonstrated in the context of X-ray imaging^17, 21^. It has been shown in ref. 21 that the quantity ${Q}_{d}^{2}={[d/(4\pi )]}^{d/2}M\,SN{R}^{2}/(\bar{n}+1/2)$ has characteristics somewhat similar to “information capacity per single particle”. Equation (8) is equivalent to the statement that ${Q}_{d}^{2}$ cannot exceed an absolute upper limit: ${Q}_{d}^{2}\le 1/{C}_{d}$.

### Experimental test of the noise-resolution uncertainty relation

**Figure 1:**
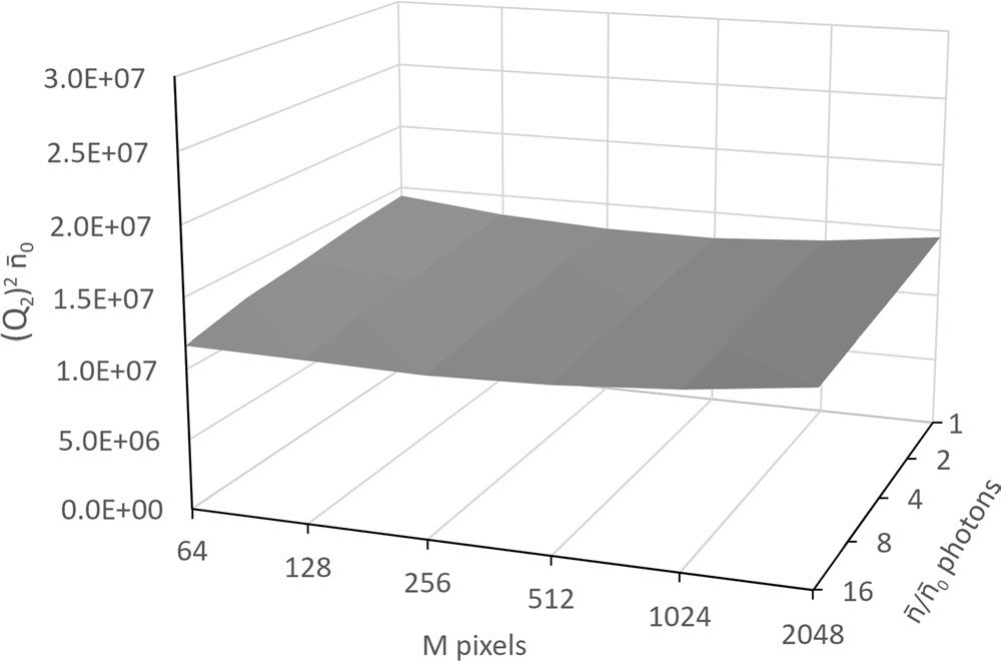
Measured dependence of the quantity ${Q}_{2}^{2}$ on the spatial resolution (number of effective pixels *M*) and on the total number of photons.

We have carried out an initial experimental verification of Equation (8) using data collected at the Imaging and Medical beamline of the Australian Synchrotron. The essential feature of this result presented in Fig. 1 is the observed approximately constant behaviour of ${Q}_{2}^{2}(M,\bar{n})$ (see details in the Methods section) within the tested range of parameters, i.e. its invariance with respect to the spatial resolution and the number of photons. According to the above theory in the case of Poissonian statistics, i.e. when *SNR* ^2^ is proportional to $\bar{n}$, one should get ${Q}_{2}^{2}(M,\bar{n})=1/C[f]$, where $C[f]=2\pi \int {|{{\bf{r}}}_{\perp }-{\bar{{\bf{r}}}}_{\perp }|}^{2}f({{\bf{r}}}_{\perp })d{{\bf{r}}}_{\perp }\int {f}^{2}({{\bf{r}}}_{\perp })d{{\bf{r}}}_{\perp }$ is a positive constant which depends only on the shape of the point-spread function, *f*(**r**_⊥_) ≡ |*u*_*k*_(**r**_⊥_)|^2^, of the detector, with **r**_**⊥**_ being a two-dimensional coordinate on the entrance surface of the detector. Therefore, the behaviour of the experimental data shown in Fig. 1 is in agreement with Equation (8) for *d* = 2. Note that, given the observed independence of ${Q}_{2}^{2}(M,\bar{n})$ from *M* and $\bar{n}$, the existence of a positive absolute lower limit in Equation (8) is then a simple consequence of the mathematical inequality (5) which holds for any function *f*(**r**_⊥_). Much more sophisticated experiments may be required in order to verify Equations (6–8) at very low photon levels, where the essentially quantum effect, such as quantum vacuum fluctuations, can have a significant impact on the results of the measurements. Such an experiment may be possible using techniques similar to those employed recently in single-particle diffraction experiments^18^ or with femtosecond laser pulses^22^.

### An extension of the Heisenberg uncertainty relation

Using the same approach, as in the derivation of Equation (6), but for the Fourier transform (momentum representation) of the mode, we obtain:

9 $${({\rm{\Delta }}p)}^{3}{({\rm{\Delta }}{\mathop{I}\limits^{ \sim }}_{\psi })}^{2}/{W}_{0}^{2}=2{\hslash }^{3}(\int d{\bf{k}}{}^{{\boldsymbol{^{\prime} }}}{|{\mathop{u}\limits^{ \sim }}_{k}({\bf{k}}{}^{{\boldsymbol{^{\prime} }}})|}^{4}\,){(\int d{\bf{k}}{}^{{\boldsymbol{^{\prime} }}}{|{\bf{k}}{}^{{\boldsymbol{^{\prime} }}}-\bar{{{\bf{k}}}^{{\rm{^{\prime} }}}}|}^{2}{|{\mathop{u}\limits^{ \sim }}_{k}({\bf{k}}{}^{{\boldsymbol{^{\prime} }}})|}^{2})}^{3/2}\ge {\hslash }^{3}{C}_{3}^{{\rm{^{\prime} }}},$$

where Δ*p* = *ħ*Δ*k*′, Δ*k* and ${\rm{\Delta }}{\tilde{I}}_{\psi }$ are defined as in Eqs (1) and (4), respectively, but with the modes *u*_*k*_(**r**) replaced by their Fourier transforms, ${\tilde{u}}_{k}({\bf{k}}{\boldsymbol{^{\prime} }})={(2\pi )}^{-3/2}\int d{\bf{r}}\,\exp \,(-i{\bf{k}}{\boldsymbol{^{\prime} }}\cdot {\bf{r}})\,{u}_{k}({\bf{r}})$, and the integration over **r** replaced with the integration over **k** *’*. Multiplying Equations (6) and (9), and taking into account the Heisenberg uncertainty inequality for *d* = 3^11^, i.e. Δ*r*Δ*p* ≥ 3*ħ*/2, we obtain the second key result of this Report - an extension of the Heisenberg uncertainty principle:

10 $${\rm{\Delta }}r\,{\rm{\Delta }}p\ge (3\hslash /2)\,{\rm{\max }}\,\{1,SN{R}_{0}[{u}_{k}]\},$$

where

11 $$SN{R}_{0}[{u}_{k}]\equiv \frac{1}{\pi }{(\frac{{C}_{3}^{2}{W}_{0}^{4}}{2{({\rm{\Delta }}{I}_{0})}^{2}{({\rm{\Delta }}{\mathop{I}\limits^{ \sim }}_{0})}^{2}})}^{1/3}=\frac{{C}_{3}^{{\rm{^{\prime} }}{\rm{^{\prime} }}}{(\int d{\bf{r}}{|{u}_{k}({\bf{r}})|}^{2})}^{4/3}}{{(\int d{\bf{r}}{|{u}_{k}({\bf{r}})|}^{4}\int d{{\bf{k}}}^{{\rm{^{\prime} }}}{|{\mathop{u}\limits^{ \sim }}_{k}({{\bf{k}}}^{{\rm{^{\prime} }}})|}^{4})}^{1/3}},$$

is a non-negative dimensionless functional defined on modes *u*_*k*_(**r**) and ${C}_{3}^{{\rm{^{\prime\prime} }}}={C}_{3}^{2/3}/(2\pi )\cong 0.14$. We included the normalization of the mode to unity into the numerator of Equation (11) to make more obvious the fact that the functional *SNR*_0_[*u*_*k*_] is bi-invariant with respect to multiplication of the mode or its argument **r** by an arbitrary positive number. That means that the value of this functional does not depend on the scaling of the height and width of the mode on which it acts, but only on its functional form. Unlike the similar bi-invariant positive functionals appearing in the Heisenberg uncertainty^11^ and in Equation (5), however, it can be shown that the value of *SNR*_0_[*u*_*k*_] can be arbitrarily large or arbitrarily close to zero for some functions, *u*_*k*_(**r**)^21^. When *SNR*_0_[*u*_*k*_] < 1, the inequality Δ*r*Δ*p* ≥ (3*ħ*/2)*SNR*_0_[*u*_*k*_] is weaker than the Heisenberg uncertainty inequality, and hence Equation (10) does not show any new effects in this instance. However, when *SNR*_0_[*u*_*k*_] > 1, inequality (10) implies that the product of uncertainties in the position and momentum of the mode has a lower limit that is larger than the one given by the Heisenberg uncertainty. In other words, inequality (10) is stronger in this case than the Heisenberg uncertainty, giving what we term an anti-squeezed Heisenberg uncertainty principle. For example, for a plane-wave mode in a cube with side length *L*, ${u}_{k}({\bf{r}})={L}^{-3/2}\exp (i{\bf{k}}\cdot {\bf{r}}){\chi }_{L}({\bf{r}})$, we obtain, $\int |{u}_{k}({\bf{r}}){|}^{4}d{\bf{r}}={L}^{-3}$, $\int |{\tilde{u}}_{k}({\bf{k}}{\boldsymbol{^{\prime} }}){|}^{4}d{\bf{k}}{\boldsymbol{^{\prime} }}={(3\pi )}^{-3}{L}^{3}$, and, hence, $SN{R}_{0}[{u}_{k}]=(3/2){C}_{3}^{2/3}\cong 1.3$ regardless of the size of the cube. Therefore, for such functions, inequality (10) already gives a larger lower bound for the minimal phase-space volume than the Heisenberg uncertainty.

Amplifying a point made earlier, when the conditions for the anti-squeezed Heisenberg uncertainty principle are met, the standard Heisenberg uncertainty principle is not violated. Rather, our results imply that the standard Heisenberg principle is not sufficiently strong under such conditions, and so its right hand side may be multiplied by a factor greater than unity. We shall discuss the physical meaning of this result at greater length later in this Report. For the moment, we note it to be quite natural that, when restricting mode functions *u*_*k*_(**r**) to a subset that has a particular value of *SNR*_0_ that is greater than unity (see Equation (11)), we have found that these modes obey a stronger form of the Heisenberg uncertainty principle in which the minimum uncertainty product is greater than that for Gaussian modes.

## Discussion

We have shown in Equation (6) that the product of the variance of electric energy of a mode and the variance of its spatial distribution, cannot be made smaller than the square of the energy of the mode in the vacuum state multiplied by a certain positive dimensionless constant. In this context, the minimum uncertainty state is achieved for Epanechnikov distributions^6^. We then demonstrated in Equation (8) that if the photon energy statistics is known, the uncertainty relation between the precision of spatial localization of the mode and the precision of its intensity measurement can be specialized and made stronger for different types of the statistics. It could be interesting to consider the possibility of extending the noise-resolution uncertainty similar to Equation (6) and Equation (8) from the energy of an electromagnetic mode to the photon number operator. One could argue that in most optical experiments it is the number of photons, rather than the field energy, that is measured by the detector^7, 20^ (note, however, the result of recent direct measurements of vacuum energy fluctuations^22^). In relation to the noise-resolution uncertainty, the key issue is that while the variance of field’s energy has a positive absolute lower limit, due to the existence of vacuum fluctuations, the variance of photon number operator can be zero, as e.g. in Fock states. Therefore, direct analogues of inequalities (2)–(3) cannot exist for the photon number operator. Nevertheless, it is also known that states with low photon number variance have weak spatial localization. Therefore, a noise-resolution uncertainty relation between the width of the spatial distribution of a mode and the variance of the number of photons can still exist, but a relevant proof will have to be different from the one used for Equation (6) above.

Now let us further consider the physical meaning of the second main result of this paper, i.e. an extension of the Heisenberg uncertainty in the form of Equation (10). It is evident from the definition of *SNR*_0_[*u*_*k*_] in Equation (11), that the case *SNR*_0_[*u*_*k*_] > 1 generally corresponds to measurements with low variance or high SNR. This result may seem counter-intuitive at first, as it states that joint high-SNR measurements of the position and momentum of an electromagnetic field must produce a very imprecise result in terms of the position and momentum localization. This is, however, a direct consequence of the noise-resolution uncertainty principle which makes the position and momentum variance larger, whenever the noise level in the corresponding measurement decreases towards its lower limit. Therefore, Equation (10) can be viewed as a logical generalization of the Heisenberg uncertainty to the case of measurements involving multiple particles (boson fields). In this case, the uncertainty in measurements of conjugate observables can be traded not only for each other, but also for the SNR in the measurement of each observable. The “non-reducible” quantity here is not the minimal phase-space volume Δ*r*Δ*p*, but the ratio of Δ*r*Δ*p* and the “phase-space vacuum SNR” of the measurements, $\max \,\{1,\,SN{R}_{0}[{u}_{k}]\}$. On the other hand, when this SNR becomes less than 1, the classical Heisenberg uncertainty takes over, with the consequence that no improvement in the accuracy of simultaneous measurements of the position and momentum beyond the Heisenberg limit can be achieved using measurements with low SNR. Of course, these conclusions have to be viewed in the context of the specific meaning of the “phase-space vacuum SNR” as defined by Equation (11).

## Methods

In the Results section above, we used simple direct calculations for the energy of the electromagnetic field in the coherent and Fock states. One could argue that more general calculations could be attempted using the formalism of *n-*point correlation functions often employed for similar purposes in quantum optics. It turns out that, for a field state with a low number of photons, the latter approach, if used without attention to relevant inherent approximations, can lead to incorrect results. It is instructive to consider this methodological issue here as it provides an insight into the nature of our central result, Equation (6). Let the first and the second order correlation functions of the field *E*(**x**) have the usual form^7, 20^:

12 $$\begin{array}{rcl}{G}^{(1)}({{\bf{x}}}_{1},{{\bf{x}}}_{2}) & = & Tr(\rho {E}^{(-)}({{\bf{x}}}_{1}){E}^{(+)}({{\bf{x}}}_{2}))\\  & = & {\sum }_{{k}_{1},{k}_{2}}{w}_{{k}_{1}}^{\ast }({{\bf{x}}}_{1}){w}_{{k}_{2}}({{\bf{x}}}_{2})\int d{\alpha }_{{k}_{1}}d{\alpha }_{{k}_{2}}P({\alpha }_{{k}_{1}},{\alpha }_{{k}_{2}})\,{\alpha }_{{k}_{1}}^{\ast }{\alpha }_{{k}_{2}}\end{array},$$

13 $$\begin{array}{ccc}{G}^{(2)}({{\bf{x}}}_{1},{{\bf{x}}}_{2},{{\bf{x}}}_{3},{{\bf{x}}}_{4}) & = & Tr(\rho {E}^{(-)}({{\bf{x}}}_{1}){E}^{(-)}({{\bf{x}}}_{2}){E}^{(+)}({{\bf{x}}}_{3}){E}^{(+)}({{\bf{x}}}_{4}))\\  & = & {\sum }_{{k}_{1},{k}_{2},{k}_{3},{k}_{4}}{w}_{{k}_{1}}^{\ast }({{\bf{x}}}_{1}){w}_{{k}_{2}}^{\ast }({{\bf{x}}}_{2}){w}_{{k}_{3}}({{\bf{x}}}_{3}){w}_{{k}_{4}}({{\bf{x}}}_{4})\\  &  & \times \int d{\alpha }_{{k}_{1}}d{\alpha }_{{k}_{2}}d{\alpha }_{{k}_{3}}d{\alpha }_{{k}_{4}}\\  &  & \times P({\alpha }_{{k}_{1}},{\alpha }_{{k}_{2}},{\alpha }_{{k}_{3}},{\alpha }_{{k}_{4}})\,{\alpha }_{{k}_{1}}^{\ast }{\alpha }_{{k}_{2}}^{\ast }{\alpha }_{{k}_{3}}{\alpha }_{{k}_{4}},\end{array}$$

where ${w}_{k}({\bf{r}},t)={v}_{k}({\bf{r}})\exp (-i{\omega }_{k}t)$, ${v}_{k}({\bf{r}})={(\hslash {\omega }_{k}/2)}^{1/2}{u}_{k}({\bf{r}})$, $\rho =\int P(\{\alpha \})|\{\alpha \}\rangle \langle \{\alpha \}|d\{\alpha \}$ is the Glauber-Sudarshan P-representation of the density operator in terms of the projections on the Glauber coherent states and $P({\alpha }_{{k}_{1}},\mathrm{...},{\alpha }_{{k}_{n}})=\int P(\{\alpha \})\prod _{\{\alpha \}\backslash \{{\alpha }_{{k}_{1}},\mathrm{...},{\alpha }_{{k}_{n}}\}}d\{\alpha \}$ are the marginal quasi-probability densities. The time-averaged space-integrated variance of the field’s intensity is then equal to

14 $${(\Delta {I}_{G})}^{2}\equiv \mathop{\mathrm{lim}}\limits_{T\to \infty }\frac{1}{T}{\int }_{-T/2}^{T/2}dt\int d{\bf{r}}\{{G}^{(2)}({\bf{r}},t,{\bf{r}},t,{\bf{r}},t,{\bf{r}},t)-{[{G}^{(1)}({\bf{r}},t,{\bf{r}},t)]}^{2}\}.$$

Substituting Equations (12) and (13) into Equation (14), we obtain:

15 $$\begin{array}{ccc}{({\rm{\Delta }}{I}_{G})}^{2} & = & \int d{\bf{r}}{\sum }_{\begin{array}{c}{k}_{1},{k}_{2},{k}_{3},{k}_{4}\\ {k}_{1}+{k}_{2}={k}_{3}+{k}_{4}\end{array}}{v}_{{k}_{1}}^{\ast }({\bf{r}}){v}_{{k}_{2}}^{\ast }({\bf{r}}){v}_{{k}_{3}}({\bf{r}}){v}_{{k}_{4}}({\bf{r}})\int d{\alpha }_{{k}_{1}}d{\alpha }_{{k}_{2}}d{\alpha }_{{k}_{3}}d{\alpha }_{{k}_{4}}\\  &  & \times {\alpha }_{{k}_{1}}^{\ast }{\alpha }_{{k}_{2}}^{\ast }{\alpha }_{{k}_{3}}{\alpha }_{{k}_{4}}[P({\alpha }_{{k}_{1}},{\alpha }_{{k}_{2}},{\alpha }_{{k}_{3}},{\alpha }_{{k}_{4}})-P({\alpha }_{{k}_{1}},{\alpha }_{{k}_{3}})P({\alpha }_{{k}_{2}},{\alpha }_{{k}_{4}})].\end{array}$$

In particular, for a state in which only a single mode, *E*_*k*_(**r**, *t*), is excited we have:

16 $${({\rm{\Delta }}{I}_{G})}^{2}={({\rm{\Delta }}{n}_{k})}^{2}{(\hslash {\omega }_{k}/2)}^{2}\,\int d{\bf{r}}|{u}_{k}({\bf{r}}){|}^{4},$$

where

17 $${(\Delta {n}_{k})}^{2}=\int d{\alpha }_{k}P({\alpha }_{k})|{\alpha }_{k}{|}^{4}-{(\int d{\alpha }_{k}P({\alpha }_{k})|{\alpha }_{k}{|}^{2})}^{2}=\,\overline{{n}_{k}^{2}}-{({\bar{n}}_{k})}^{2}$$

is the variance of the number of photons in the mode. Depending on the quasi-probability density distribution *P*(*α*), the value of (Δ*n*_*k*_)^2^ in Equation (17) can be can be close to $\overline{{n}_{k}^{2}}$ or to zero. In particular, for a pure coherent state, we have *P*(*α*) = *δ*(*α*−*α*_0_) and hence ${({\rm{\Delta }}{n}_{k})}^{2}=0.$ Obviously, this is a non-physical result, as the intensity measurement cannot have zero variance even for a pure coherent state^20^. This “paradox” appears because the above approach to calculation of field correlations neglects the commutators of the photon annihilation and creation operators which arise during the process of reduction of the expressions for correlation functions to a normally ordered form. The approximation usually works well for states with large numbers of photons, but it can be inaccurate when the number of photons is low. In particular, the contribution of vacuum fluctuations is ignored as a consequence of this approximation, which explains the difference between Equations (16) and (17) and the exact results for the Fock and coherent states presented in Equations (2) and (3) above. Note, however, that the consequences of neglecting the commutator $[a,{a}^{\dagger }]$ are not limited to the omission of vacuum fluctuations. Indeed, if one calculates the variance of the photon number operator $\hat{n}={a}^{\dagger }a$ in a coherent state |α〉 using normal ordering and ignoring the commutators, i.e. approximating ${({a}^{\dagger }a)}^{2}$ with ${({a}^{\dagger })}^{2}{a}^{2}$ in the first term on the right-hand side of the expression ${(\Delta {n}_{k})}^{2}=\langle \alpha |{({a}^{\dagger }a)}^{2}|\alpha \rangle -{(\langle \alpha |{a}^{\dagger }a|\alpha \rangle )}^{2}$, one would get (Δ*n*_*k*_)^2^ = 0, instead of the correct answer (Δ*n*_*k*_)^2^ = |*α*|^2^. This is consistent with the above “paradox” appearing in Equations (16) and (17), and explains why we had to use direct calculations of the field’s energy and its variance, instead of relying on generic quantum optical correlation functions.

In our experimental test, we have collected 1024 images of an unobstructed nearly-parallel wide monochromatic X-ray beam with energy of 30 keV using a Hamamatsu CMOS Flat Panel detector C9252DK-14 in “partial field” mode with pixel size 100 microns. We subsequently selected a uniformly illuminated region with 8 (vertical) × 256 (horizontal) pixels inside which we measured the average intensity and its variance. By binning pixels in the horizontal direction by the factors 2^*j*^, *j* = 0,1,..., 5, we were able to systematically vary the effective pixel size and, hence, the spatial resolution in the images. By adding image frames together into bunches of 1, 2, 4, 8 and 16 frames, we were able to independently vary the number of photons in each pixel. As the actual number of registered photons was unknown (because of the difficulties in measuring the detective quantum efficiency of the detector with sufficient accuracy), we present the output data as a function of the relative number of photons, $\bar{n}/{\bar{n}}_{0}$, where ${\bar{n}}_{0}$ is an unknown constant equal to the average total number of photons in a single image frame. Figure 1 depicts the obtained dependence of the quantity ${\bar{n}}_{0}{Q}_{2}^{2}(M,\bar{n})=\frac{M\,SN{R}^{2}\,{\bar{n}}_{0}}{2\pi (\bar{n}+1/2)}=\frac{{L}^{2}}{2\pi {[{\rm{\Delta }}r(M)]}^{2}}\frac{{I}_{L,\psi }^{2}}{{({\rm{\Delta }}{I}_{L,\psi })}^{2}}\frac{{\bar{n}}_{0}}{(\bar{n}+1/2)}$ on spatial resolution (number of effective pixels *M*) and on the average total number of photons, $\bar{n}$.
